# Association of liver dysfunction with outcomes after percutaneous coronary intervention – a systematic review and meta-analysis

**DOI:** 10.1186/s12872-024-04252-9

**Published:** 2024-10-21

**Authors:** Tulio Caldonazo, Hristo Kirov, Panagiotis Tasoudis, Marcus Franz, Markus Richter, Murat Mukharyamov, Alexander Zipprich, Ahmad Shana’ah, Diyar Haji, Torsten Doenst

**Affiliations:** 1 Department of Cardiothoracic Surgery, Jena University Hospital, Friedrich-Schiller-University, University of Jena, Am Klinikum 1, 07747 Jena, Germany; 2 Division of Cardiothoracic Surgery, University of North Carolina, Chapel Hill, USA; 3 Division of Cardiology, Department of Internal Medicine, Jena University Hospital, Friedrich-Schiller-University Jena, Jena, Germany; 4 Department of Internal Medicine IV (Gastroenterology, Hepatology, Infectiology), Jena University Hospital, Friedrich-Schiller-University Jena, Jena, Germany

**Keywords:** Liver dysfunction, Percutaneous coronary intervention, Coronary artery disease

## Abstract

**Background:**

Liver dysfunction is a known risk factor in the cardiovascular field. It specifically increases perioperative risk in patients undergoing coronary bypass surgery. Since percutaneous coronary intervention (PCI) is the much less invasive procedure for the treatment of coronary artery disease, we aimed to assess the relationship of liver dysfunction with outcomes in patients undergoing PCI.

**Methods:**

Three libraries were searched (MEDLINE, Web of Science and The Cochrane Library). We performed a meta-analysis of all studies in patients who underwent PCI that provided information on the presence or absence of liver dysfunction. Primary outcome was short-term mortality. Secondary outcomes were major adverse cardio- and cerebrovascular events (MACCE), bleeding and acute kidney injury. Random-effects model was applied.

**Results:**

Five studies were selected and the data from 10,710,317 patients were included in the final analysis. In comparison with the absence of liver dysfunction, patients with liver dysfunction were associated with higher short-term mortality (OR 2.97, 95%CI 1.23–7.18, *p* = 0.02), higher MACCE (OR 1.42, 95%CI 1.08–1.87, *p* = 0.01), and higher bleeding (OR 2.23, 95%CI 1.65-3.00, *p* < 0.01). There was no significant difference regarding acute kidney injury (OR 1.20, 95%CI 0.50–2.87, *p* = 0.69).

**Conclusions:**

The analysis suggests that liver dysfunction in patients undergoing PCI is independently associated with higher risk of short-term mortality and increased occurrence of MACCE and bleeding. However, there appears to be no association to acute kidney injury.

**Supplementary Information:**

The online version contains supplementary material available at 10.1186/s12872-024-04252-9.

## Introduction

The global burden of liver disease has been rising over the last decades and currently liver dysfunction accounts for approximately two million deaths per year worldwide [1]. About 75 million are at risk of the classic, alcohol-associated liver disease [1] but the global prevalence non-alcoholic fatty liver disease (NAFLD) is much larger amounting to approximately two billion people affected worldwide [2]. Importantly, cardiovascular disease (CVD) represents the most common cause of death in patients with NAFLD so that the European clinical practice guidelines for management of NAFLD recommend mandatory CVD screenings in these patients [3]. Furthermore, liver dysfunction may be a result of not only NAFLD or alcoholic liver disease but may derive from a wide spectrum of other diseases mainly virus hepatitis, autoimmune disorders and hereditary diseases. Thus, the overall number of patients with liver dysfunction and concomitant coronary artery disease might be even higher, with a great number of them requiring interventional diagnosing and therapy.

Liver dysfunction has already been recognized as a risk factor in cardiac surgery [4] and has been even included in the preoperative assessment of surgical risk (e.g. STS-score [5]). In comparison to coronary artery bypass grafting (CABG), percutaneous coronary interventions (PCI) represent a less-invasive alternative to treat coronary artery disease [6, 7], but a thorough assessment of liver dysfunction on mortality and other peri-interventional complications with PCI has not been published, yet.

Thus, we aimed to evaluate the impact of liver dysfunction in PCI, summarizing the existing body of scientific publications. We therefore set out to systematically review the literature and thoroughly assess the effect of liver dysfunction by way of a meta-analysis on clinical outcome after PCI, with a focus on short-term mortality and major adverse cardio- and cerebrovascular events (MACCE) as well as bleeding and acute kidney injury.

## Methods

Ethical and IRB approval of this analysis were not required as no human or animal subjects were involved. This review was registered with the National Institute for Health Research International Registry of Systematic Reviews (PROSPERO, CRD42022329323).

### Search strategy

A comprehensive literature search to identify contemporary studies reporting short- and long-term outcomes in patients with liver dysfunction compared to patients with no or mild liver dysfunction who underwent percutaneous coronary intervention. Searches were run on April 2022 in the following databases: Ovid MEDLINE^®^, Ovid Web of Science and The Cochrane Library. The search strategy for Ovid MEDLINE is available in Supplementary Table 1.

### Study selection and data extraction

The study selection followed the Preferred Reporting Items for Systematic Reviews and Meta-Analyses (PRISMA) strategy. After de-duplication, records were screened by two independent reviewers (AS and DH). Any discrepancies and disagreements were resolved by a third author (TD). Titles and abstracts were reviewed against pre-defined inclusion and exclusion criteria. Studies were considered for inclusion if they were written in English and reported direct comparison between patients with liver dysfunction and patients with no or mild liver dysfunction underwent percutaneous coronary intervention. The severity of liver dysfunction was evaluated with preoperative Model for End-Stage Liver Disease (MELD) score and fatty liver disease (FLD) score [8, 9]. Patients were stratified in the liver dysfunction group and in the no/mild liver dysfunction based on the cutoffs proposed by each study as previously published [10–12]. Animal studies, abstracts, case reports, commentaries, editorials, expert opinions, conference presentations, and studies not reporting the outcomes of interest were excluded. The full text was pulled for the selected studies for a second round of eligibility screening. References for articles selected were also reviewed for relevant studies not captured by the original search.

The quality of the included studies was assessed using the Newcastle-Ottawa Scale for observational studies (Supplementary Table 2) [13].

Two reviewers (AS and DH) independently performed data extraction, and the accuracy was verified by a third author (TD). The variables included were: study characteristics (publication year, country, sample size, and type of surgery), patient demographics (age, sex, hypertension, diabetes mellitus, prior percutaneous coronary intervention [PCI], prior cerebrovascular accident [CVA], and chronic renal failure).

### Outcomes

The primary outcome was short-term mortality (< 90-days). The secondary outcomes were MACCE, bleeding and acute kidney injury. Supplementary Tables 3 and 4 show the criteria used to divide the study populations and the individual study outcomes definitions, respectively.

### Statistical analysis

We conducted meta-analyses to compare the outcomes of no/mild liver dysfunction versus liver dysfunction groups. Briefly, absolute values regarding the number of events and the total population were extracted. These data were compiled and odds ratio (OR) of individual studies was calculated. Random effects DerSimonian-Laird model was performed to pool the data and provide the final treatment effect. The overall OR and 95% confidence intervals (CI) were calculated for each outcome. A OR greater than 1 indicated that the outcome was more frequently present in the liver dysfunction arm. Inherent clinical heterogeneity between the studies was balanced via the implementation of a random effects models [14, 15]. Results were displayed in forest plots. Between-study statistical heterogeneity was assessed with the Cochran Q statistic and by estimating I^2^. High heterogeneity was confirmed with a significance level of *p* < 0.10 and I2 of at least 50% or more. Publication bias was assessed via funnel plots and Eggers’ test for the primary outcome and *p* < 0.10 was considered statistically significant. Leave-one-out sensitivity analysis and a funnel plot were also generated for the primary outcome [15]. All analyses were performed using STATA IC17.0 (StataCorp LLC, College Station, Texas).

## Results

### Study and patient characteristics

**Fig. 1:**
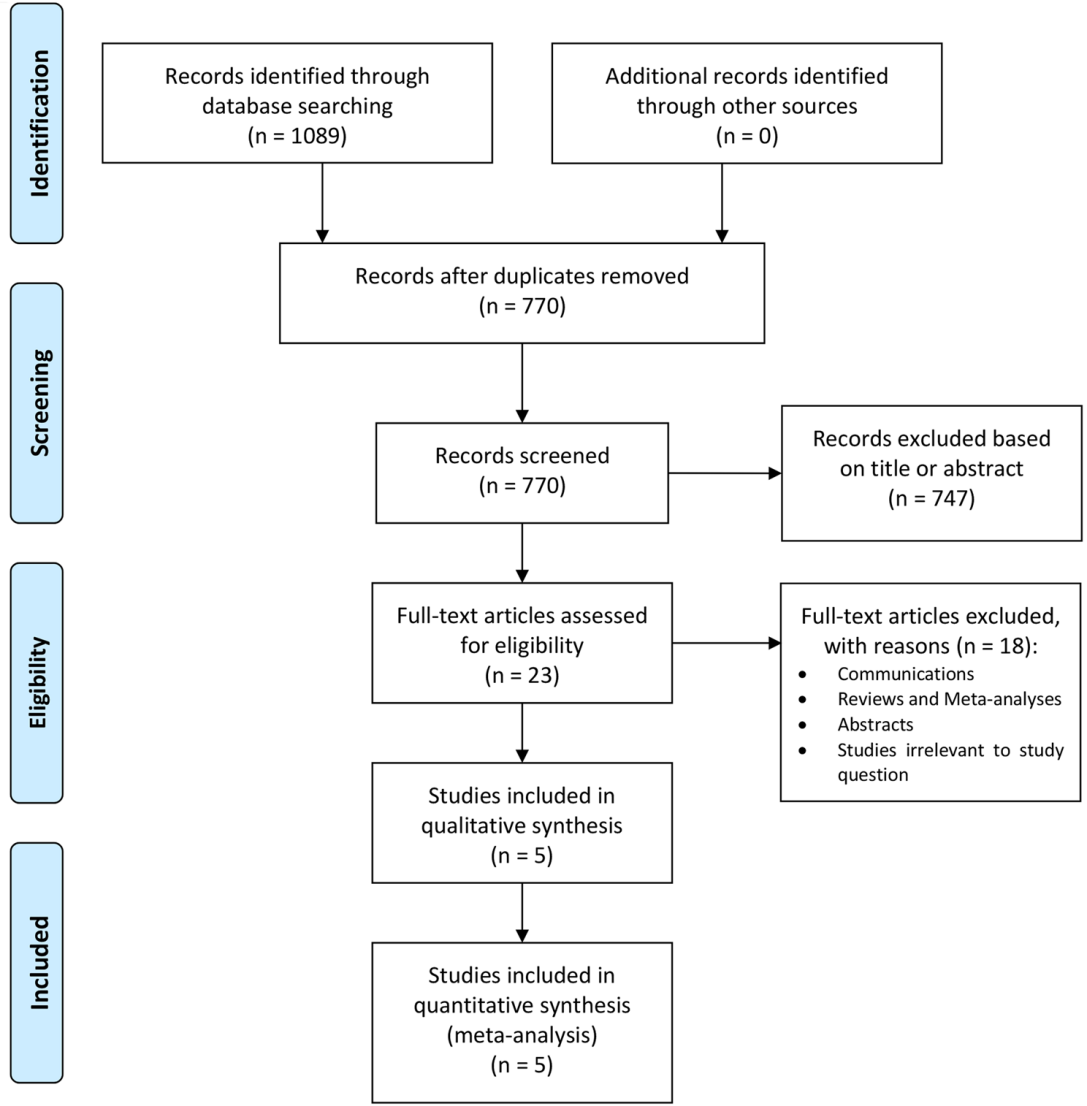
Preferred reporting items for systematic reviews and meta-analyses (PRISMA) flow diagram

A total of 1,089 studies were retrieved from the systematic search, of which 5 met the criteria for inclusion in the final analysis. The PRISMA flowchart for study selection is provided in Fig. 1. Included studies were published between 2015 and 2021, all were observational risk-adjusted cohort studies, and 3 were multi-center. Three originated from national registries from the United States, 1 from Turkey and 1 from China. Details of the included studies are provided in Table 1.

**Table 1:** Summary of included studies

Author	Year of Publication	Country	*N*° of patients	Study Design	Follow-up	Population Comparability	Reported Outcomes
Emre [16]	2015	Turkey	186	Prospective, single center	In-hospital outcomes	Multivariable logistic regression	• Mortality • MACCE
Alqahtani [32]	2019	United States	8,860,178	Retrospective, multicenter	In-hospital outcomes	Propensity score-matching	• Acute kidney injury
Lu [33]	2020	United States	1,844,580	Retrospective, multicenter	3 months	Multivariable logistic regression	• Mortality • Neurological event • Acute kidney injury • Bleeding
Instabuly [34]	2021	United States	7,296,679	Retrospective, multicenter	In-hospital outcomes	Multivariable logistic regression	• Mortality • Neurological event • Bleeding • MACCE
Chen [31]	2021	China	5,373	Retrospective, single center	10 years	Multivariable cox regression	• Bleeding • MACCE

PCI = percutaneous coronary intervention, MACCE = major adverse cardio- and cerebrovascular events

Data from 10,710,317 patients were included in the final analysis. Demographic data of the patient population in each study are summarized in Supplementary Table 5. The mean age ranged from 60.0 to 64.0 years in the liver dysfunction group and from 56.0 to 65.0 in the no liver dysfunction group. The percentage of male patients ranged from 68.4 to 84.2 in the liver dysfunction group and from 64.8 to 79 in the no liver dysfunction group. The prevalence of hypertension ranged from 43.5 to 72.2 in the liver dysfunction group and from 42.5 to 74.3 in the no liver dysfunction group. The prevalence of diabetes mellitus ranged from 23.2 to 51.3 in the liver dysfunction group and from 25.6 to 36.7 in the no liver dysfunction group. The prevalence of prior cerebrovascular events ranged from 0.9 to 3.2 in the liver dysfunction group and from 1.8 to 3.5 in the no liver dysfunction group. The prevalence of prior myocardial infarction ranged from 13.2 to 14.7 in the liver dysfunction group and from 13.3 to 13.9 in the no liver dysfunction group. The prevalence of chronic renal failure ranged from 17.1 to 28 in the liver dysfunction group and from 9.9 to 13.4 in the no liver dysfunction group.

### Meta-analysis

**Fig. 2:**
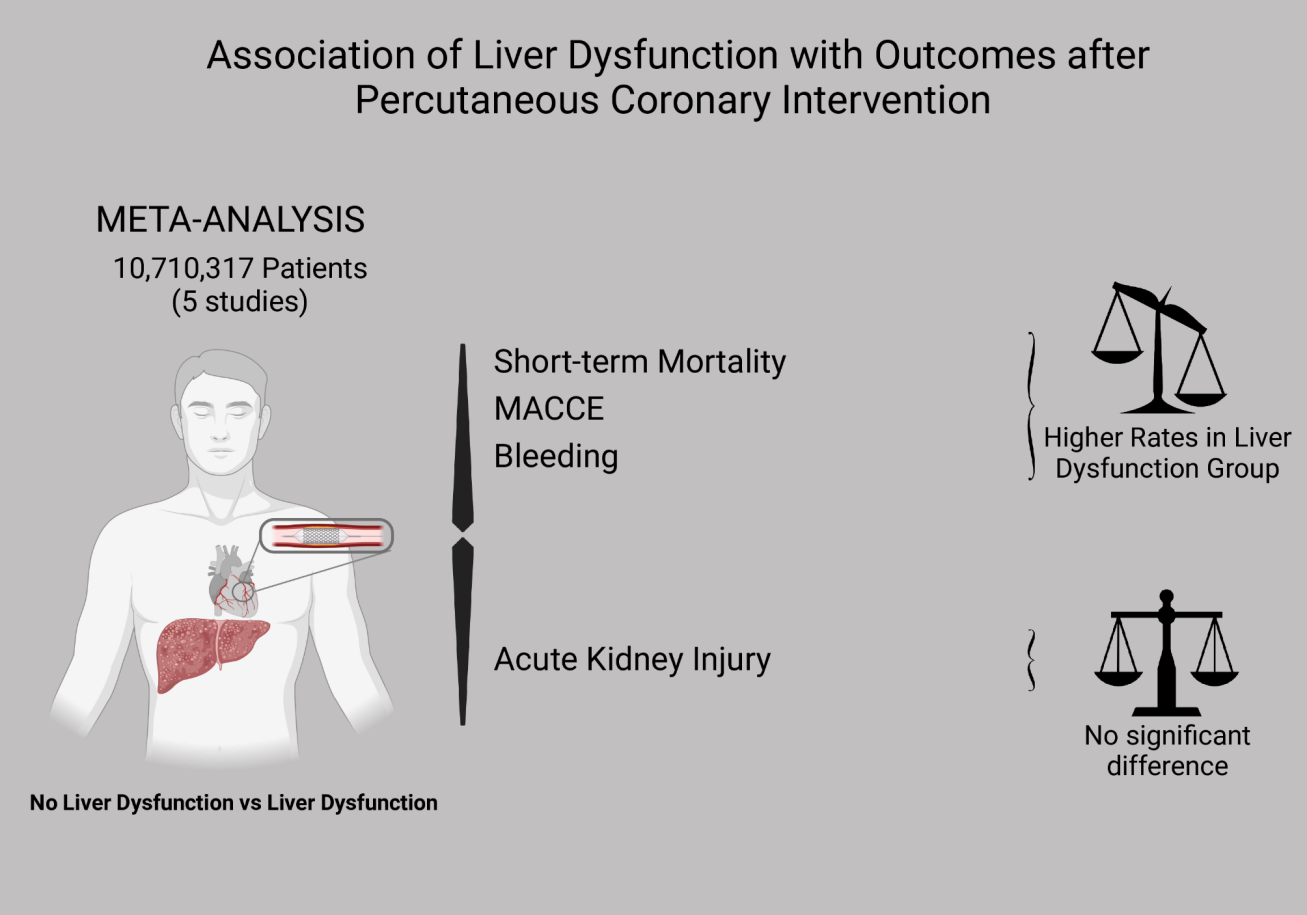
(Central Picture). Graphical abstract showing the main findings of the analysis. MACCE = major adverse cardio- and cerebrovascular event

Figure 2; Table 2 outline the detailed results of the meta-analysis.

**Table 2:** Outcomes summary

Outcome	Number of Studies	Number of Patients	Effect Estimate (95%CI, *p*-value)
Short-term mortality	3	9,141,445	2.97 (1.23–7.18, *p* = 0.02)
Major adverse cardiovascular events	3	7,302,238	1.42 (1.08–1.87, *p* = 0.01)
Bleeding	3	9,146,632	2.23 (1.65-3.00, *p* < 0.01)
Acute kidney injury	4	1,885,076	1.20 (0.50–2.87, *p* = 0.69)

OR = odds ratio

### Primary outcome

**Fig. 3:**
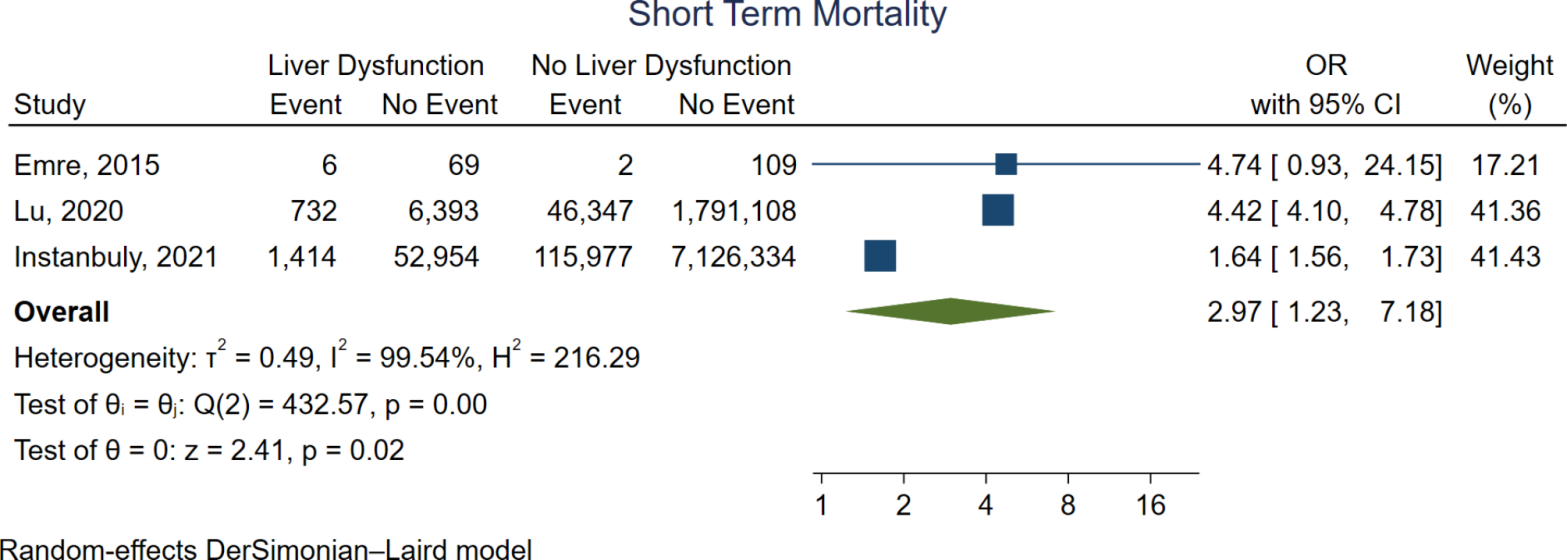
Forest plot for short-term mortality. CI = confidence interval, OR = odds ratio

Figure 3 shows the forest plot for short-term mortality. Three out of the five studies reported short-term mortality. Compared with no liver dysfunction group, the liver dysfunction group was associated with significantly higher short-term mortality (OR 2.97, 95%CI 1.23–7.18, *p* = 0.02).

Supplementary Fig. 1 shows the leave-one-out analysis, which confirmed the robustness of the main analysis. One study used the FLD Score for the assessment of liver dysfunction [16] while the other selected patients with diagnosed liver cirrhosis. Since there is debate about the value of the FLD score [17], patients with a high FLD score may not have significant liver dysfunction. However, the omission of this study from the analysis did not alter the main outcome. Liver dysfunction remained associated with higher short-term mortality (OR 2.69, 95% CI, 1.02–7.12, *p* = 0.04). Supplementary Fig. 2 shows the funnel plot.

### Secondary outcomes

**Fig. 4:**
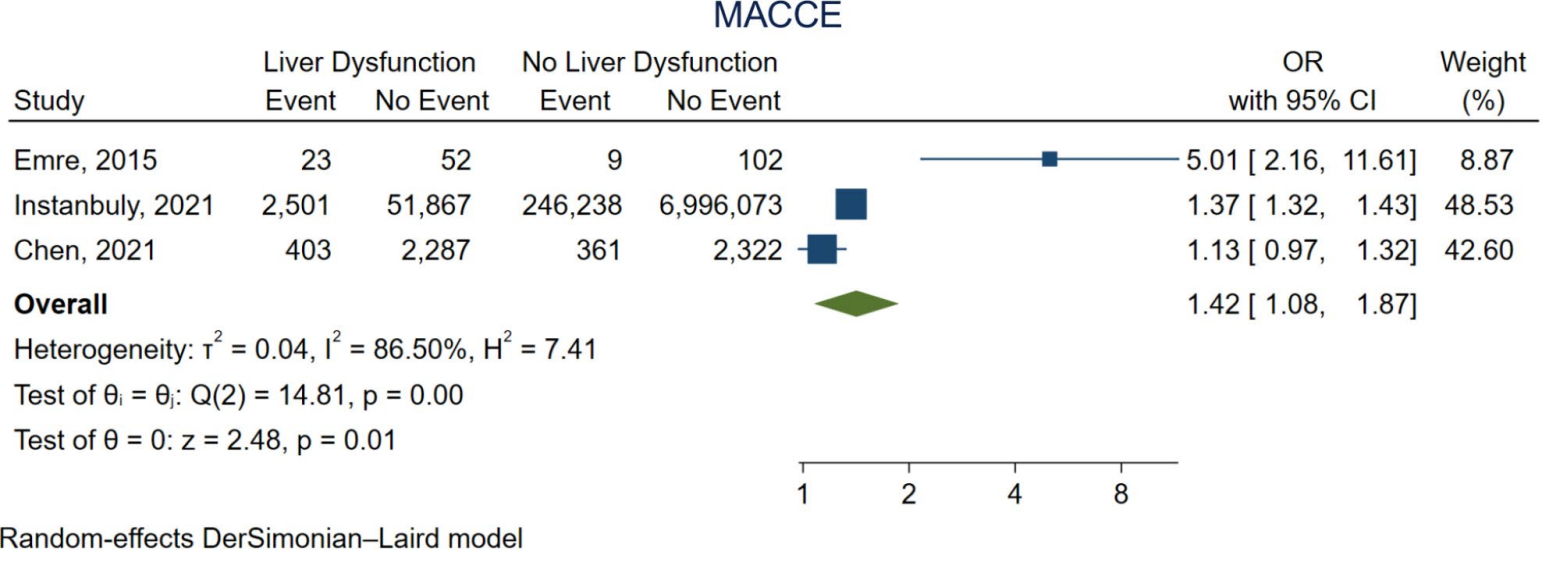
Forest plot for major adverse cardiovascular events. CI = confidence interval, OR = odds ratio

Figure 4 shows the forest plot for MACCE. The presence of liver dysfunction was associated with significantly higher MACCE (OR 1.42, 95%CI 1.08–1.87, *p* = 0.01).

**Fig. 5:**
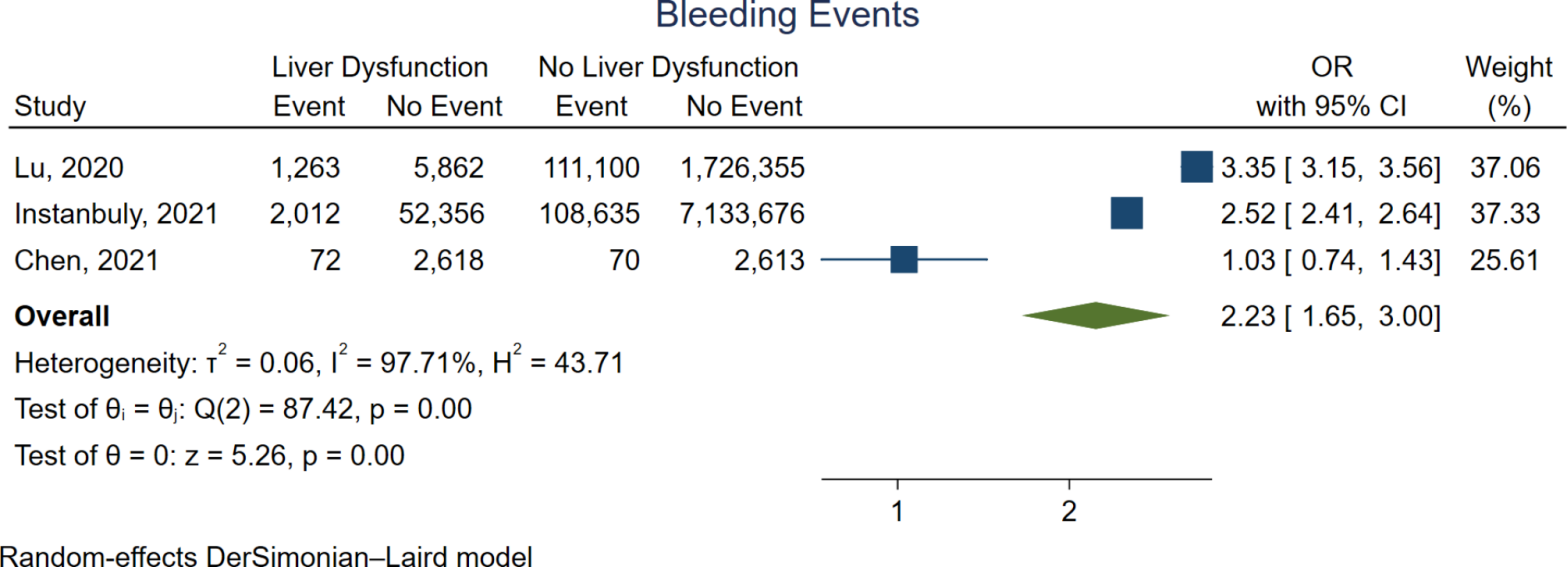
Forest plot for bleeding. CI = confidence interval, OR = odds ratio

Figure 5 shows the forest plot for bleeding. The presence of liver dysfunction was associated with significantly higher bleeding rates (OR 2.23, 95%CI 1.65-3.00, *p* < 0.01).

**Fig. 6:**
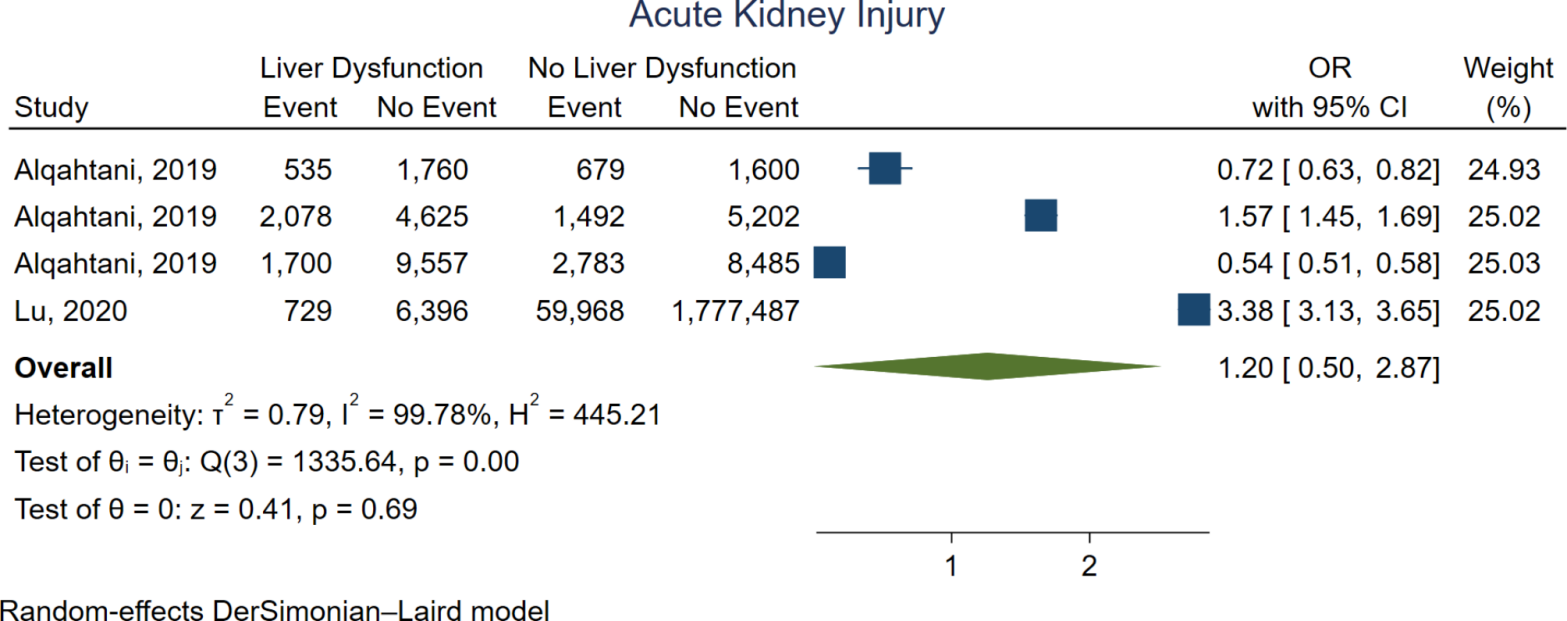
Forest plot for acute kidney failure. CI = confidence interval, OR = odds ratio

Figure 6 shows the forest plot for acute kidney injury. There was no significant difference between the groups (OR 1.20, 95%CI 0.50–2.87, *p* = 0.69). Yet, one study subdivided its data based on urgency and found more renal failure with higher urgency of the operation.

## Discussion

We demonstrate in this analysis that liver dysfunction in patients undergoing percutaneous coronary intervention is independently associated with higher risk of short-term mortality and with an increased occurrence of MACCE and bleeding. However, there appears to be no association of liver dysfunction with acute kidney injury after PCI.

Our results are relevant as the evaluation of various risk factors and their association with outcomes is an important aspect of clinical decision-making. This is especially important in the treatment of coronary artery disease (CAD), where the therapeutic decisions (surgery vs. intervention vs. conservative medical therapy), might be based on the presence or absence of risk factors (e.g., diabetes, chronic kidney disease, frailty), [18, 19]. There is an ongoing debate whether CABG or PCI provide better results for the treatment of CAD [6, 20, 21]. We showed in a recent analysis that liver dysfunction in cardiac surgery is associated with higher rates of neurological events, prolonged ventilation, sepsis, bleeding, acute kidney injury, short and long-term mortality [4].

As PCI is much less-invasive than CABG, we speculated that liver dysfunction would not affect short-term outcomes of PCI, because perioperative risk is thought to be associated with surgical trauma and/or the use of cardiopulmonary bypass [22]. From that perspective, the strong association of liver dysfunction with short term-mortality, MACCE and bleeding in PCI may be surprising. However, quantitative comparisons of the impact of liver dysfunction on outcomes between CABG and PCI are not possible from these analyses. Interestingly, liver dysfunction is associated with acute kidney injury in surgery [23], but not in PCI, which may be relevant for decision-making, specifically in patients with liver dysfunction and already impaired kidney function before an invasive procedure. However, the retrospective nature of the studies included in this report makes them burdened with various biases (incl. selection, surgeon/interventionalist, publication or investigator biases) which preclude direct conclusions guiding treatment recommendations. Yet, the data provide important and hypothesis-generating evidence, which calls for a randomized controlled trial comparing CABG and PCI for patients with liver dysfunction.

Another important aspect of our analysis is the large number of included patients (more than 10 million), which additionally strengthens our findings and limits the probability of random error occurrence or overestimation of the results [24]. It is interesting to note that despite this large body of evidence, prediction of short-term complications of PCI is not a recommended or emphasized part of pre-procedural patient workup, specifically not of the informed consent process. In contrast, the guidelines suggest to assess the suitability of PCI based on the SYNTAX score and recommend assessing surgical risk to predict potential complications with the surgical procedure. Decision trees are then provided only for patients with low surgical risk [18], indirectly suggesting that patients with high surgical risk that have a PCI suitable anatomy are immediately candidates for PCI. Given the findings of this meta-analysis, patients with liver dysfunction are also at high risk if it comes to PCI. It might be similarly surprising, that direct comparisons of short-term mortality after PCI and CABG (without specific focus on liver disease) found identical values at 30 days in randomized [25] and non-randomized [20, 26] studies. Thus, it may be time to apply risk prediction models also for PCI [27, 28]. From this study’s perspective they would also have to include liver dysfunction, which is currently not the case [27, 28].

Appropriately assessing peri-interventional risk is important, specifically if PCI is the primary treatment option (e.g., ST-elevation myocardial infarction). In such cases, being aware of all relevant risk factors may help in defining a suitable invasive strategy. For example, Singh et al. [29] identified in a propensity-matched analysis of over one million PCIs that the use of bare-metal stents in patients with liver cirrhosis was associated with increased mortality (two-fold) and bleeding complications compared with drug-eluting stents.

We also found a twofold increase in bleeding risk in patients with liver dysfunction undergoing PCI in this meta-analysis. Although we analyzed studies of interventional treatment only, it might be well conceivable that this risk might be also increased in interventions with strictly diagnostic purpose. In such cases, alternative diagnostic strategies (e.g., coronary CT-scan) might be a suitable option. Regarding the anti-thrombotic status from patients with liver dysfunction, a recent study presented results that indicated that liver fibrosis scores can predict an elevated risk of thrombotic events but not bleeding [30].

To the best of our knowledge, this comprehensive meta-analysis is the first to address this important topic of liver dysfunction in the context of PCI, both in acute and elective settings and provide an overview of several clinical outcomes and their association with liver dysfunction after PCI. The findings are relevant because an increasing number of patients undergoing PCI have liver dysfunction. The results of our analysis provide a previously unknown quantification of the relationship between liver dysfunction and PCI complications.

### Study strength and limitations

This is the first meta-analysis with 4 different endpoints that compiled data from patients with hepatic dysfunction who underwent PCI. This work has the intrinsic limitations of observational series, including the risk of methodological heterogeneity of the included studies and residual confounders. Moreover, two studies provided a much larger number of patients than the others. Finally, one study used the MELD score for the assessment of liver dysfunction [31], which may not necessarily reflect the explicit diagnosis of liver disease.

## Conclusion

We demonstrate in this analysis that liver dysfunction in patients undergoing percutaneous coronary intervention is independently associated with higher risk of short-term mortality and with an increased occurrence of MACCE and bleeding. However, there appears to be no association of liver dysfunction with acute kidney injury after PCI.

## Electronic supplementary material

Below is the link to the electronic supplementary material.

Supplementary Material 1

## Data Availability

The data underlying this article are available in the article and in its online supplementary material.
