# Supplementary material for: Association of liver dysfunction with outcomes after percutaneous coronary intervention – a systematic review and meta-analysis
Source: BMC Cardiovasc Disord. 2024 Oct 21;24:580. doi: 10.1186/s12872-024-04252-9 (PMC11492513; doi:10.1186/s12872-024-04252-9)
Supplement: Supplementary file 1 — Supplementary Material 1 [file 12872_2024_4252_MOESM1_ESM.docx]

**Supplementary Material**

**Supplementary Table 1**: Search strategy for Ovid MEDLINE.

**Supplementary Table 2**: Assessment of risk of bias using the Newcastle Ottawa Scale.

**Supplementary Table 3:** Criteria used to divide the study populations.

**Supplementary Table 4**: Definition of the outcomes of interest by study.

**Supplementary Table 5**: Demographics of included patients.

**Supplementary Figure 1**: Leave-one-out analysis for short-term mortality.

**Supplementary Figure 2.** Funnel plot for short-term mortality.

**Supplementary Table 1:** Search strategy for Ovid MEDLINE.

| Search 1: **(percutaneous coronary intervention) AND ((liver dysfunction) OR (cirrhosis))** Filters: **English**  (("percutaneous coronary intervention"[MeSH Terms] OR ("percutaneous"[All Fields] AND "coronary"[All Fields] AND "intervention"[All Fields]) OR "percutaneous coronary intervention"[All Fields]) AND ("liver diseases"[MeSH Terms] OR ("liver"[All Fields] AND "diseases"[All Fields]) OR "liver diseases"[All Fields] OR ("liver"[All Fields] AND "dysfunction"[All Fields]) OR "liver dysfunction"[All Fields] OR ("liver cirrhosis"[MeSH Terms] OR ("liver"[All Fields] AND "cirrhosis"[All Fields]) OR "liver cirrhosis"[All Fields] OR "cirrhosis"[All Fields] OR "fibrosis"[MeSH Terms] OR "fibrosis"[All Fields]))) AND (english[Filter]) |
| --- |
| **Translations** |
| **percutaneous coronary intervention:** "percutaneous coronary intervention"[MeSH Terms] OR ("percutaneous"[All Fields] AND "coronary"[All Fields] AND "intervention"[All Fields]) OR "percutaneous coronary intervention"[All Fields]  **liver dysfunction:** "liver diseases"[MeSH Terms] OR ("liver"[All Fields] AND "diseases"[All Fields]) OR "liver diseases"[All Fields] OR ("liver"[All Fields] AND "dysfunction"[All Fields]) OR "liver dysfunction"[All Fields]  **cirrhosis:** "liver cirrhosis"[MeSH Terms] OR ("liver"[All Fields] AND "cirrhosis"[All Fields]) OR "liver cirrhosis"[All Fields] OR "cirrhosis"[All Fields] OR "fibrosis"[MeSH Terms] OR "fibrosis"[All Fields] |

**Supplementary Table 2:** Assessment of risk of bias using the Newcastle Ottawa Scale.

| STUDY | SELECTION | COMPARABILITY | OUTCOME/ EXPOSURE |
| --- | --- | --- | --- |
| Emre, 2015 | **** | ** | *** |
| Alqahtani, 2019 | **** | ** | *** |
| Lu, 2020 | **** | ** | *** |
| Instanbuly, 2021 | **** | ** | *** |
| Chen, 2021 | **** | ** | *** |

**Supplementary Table 3:** Criteria used to divide the study populations (no liver dysfunction and liver dysfunction).

| STUDY | CRITERIA |
| --- | --- |
| Emre, 2015 | FLD score<3 versus FLD score>3 |
| Alqahtani, 2019 | Cirrhosis based on the International Classification of Diseases, Ninth Revision − Clinical Modification (ICD-9-CM) |
| Lu, 2020 | Cirrhosis based on the International Classification of Diseases, Ninth Revision − Clinical Modification (ICD-9-CM) |
| Instanbuly, 2021 | Cirrhosis based on the International Classification of Diseases, Ninth Revision − Clinical Modification (ICD-9-CM) |
| Chen, 2021 | MELD quartile 1+2 versus MELD quartile 3+4 |

FLD: fatty liver disease, MELD= model of end-stage liver disease.

**Supplementary Table 4**: Definition of the outcomes of interest by study.

| STUDY | NEUROLOGICAL  EVENT | TRANSFUSION/ BLEEDING | MAJOR ADVERSE CARDIO- AND CEREBROVASCULAR EVENTS |
| --- | --- | --- | --- |
| Emre, 2015 | Not reported | Not reported | Nonfatal myocardial infarction, acute heart failure and mortality |
| Alqahtani, 2021 | Not reported | Not reported | Not reported |
| Lu, 2020 | Stroke | Total bleeding | Not reported |
| Instanbuly, 2021 | Stroke | Major bleeding | Mortality, major bleeding, cardiac complications and acute ischemic stroke |
| Chen, 2021 | Not reported | Bleeding events | Mortality, myocardial reinfarction, target vessel reconstruction and stroke |

**Supplementary Table 5:** Demographics of included patients.

| Study | Age (mean±SD) | | Male (%) | | | HPT (%) | | | DM (%) | | | Prior PCI (%) | | | Prior CVA (%) | | | Prior MI (%) | | | Chronic renal failure (%) | | |  |
| --- | --- | --- | --- | --- | --- | --- | --- | --- | --- | --- | --- | --- | --- | --- | --- | --- | --- | --- | --- | --- | --- | --- | --- | --- |
|  | **LD** | **No LD** | | **LD** | **No LD** | | **LD** | **No LD** | | **LD** | **No LD** | | **LD** | **No LD** | | **LD** | **No LD** | | **LD** | **No LD** | | **LD** | **No LD** | |
| Emser, 2015 | 60±11 | 56±10 | | 75 | 79 | | 51 | 59 | | NR | NR | | NR | NR | | NR | NR | | NR | NR | | NR | NR | |
| Alqahtani, 2019 - STEMI | 62±11 | 62±13 | | 74.2 | 70.7 | | 57.8 | 60.9 | | 36.3 | 28.1 | | NR | NR | | 0.9 | 1.8 | | NR | NR | | NR | NR | |
| Alqahtani, 2019 - NSTEMI | 64±10 | 65±13 | | 70.9 | 64.8 | | 67.9 | 72.1 | | 47.8 | 36.7 | | NR | NR | | 3.1 | 3.5 | | NR | NR | | NR | NR | |
| Alqahtani, 2019 – UA/SIHID | 63±12 | 65±12 | | 75 | 66.4 | | 63.3 | 69.8 | | 42.6 | 33.2 | | NR | NR | | 3.2 | 2.5 | | NR | NR | | NR | NR | |
| Lu, 2020 | 63.9 | 64.7 | | 69.4 | 67.1 | | 72.2 | 74.3 | | 51.3 | 36.4 | | 18.7 | 20.4 | | NR | NR | | 14.7 | 13.9 | | 28 | 13.4 | |
| Instanbuly, 2021 | 60±3.7 | 65±4.5 | | 68.4 | 66.4 | | 70.1 | 69.9 | | 37.5 | 29.3 | | 12.2 | 16.5 | | 2.3 | 2.2 | | 13.2 | 13.3 | | 17.1 | 9.9 | |
| Chen, 2021 | 60.2±10 | 58.8±10 | | 84.2 | 67.9 | | 43.5 | 42.5 | | 23.2 | 25.6 | | NR | NR | | NR | NR | | NR | NR | | NR | NR | |

CVA: cerebrovascular accident, DM: diabetes mellitus, HPT: hypertension, LD: liver dysfunction, MI: myocardial infarction, NR: not reported, NSTEMI: non-ST elevation myocardial infarction, PCI: percutaneous coronary intervention, SIHD: stable ischemic heart disease, SD: standard deviation, STEMI: ST-elevation myocardial infarction, UA: unstable angina.

**Supplementary Figure 1:** Leave-one-out analysis for short-term mortality.

**
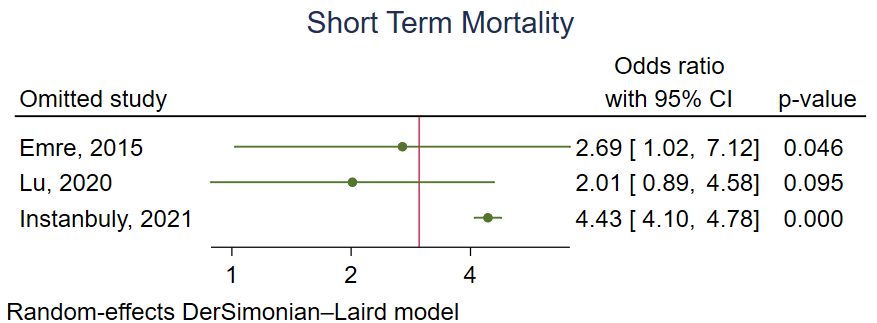
**

**Supplementary Figure 2.** Funnel plot for short-term mortality.

**
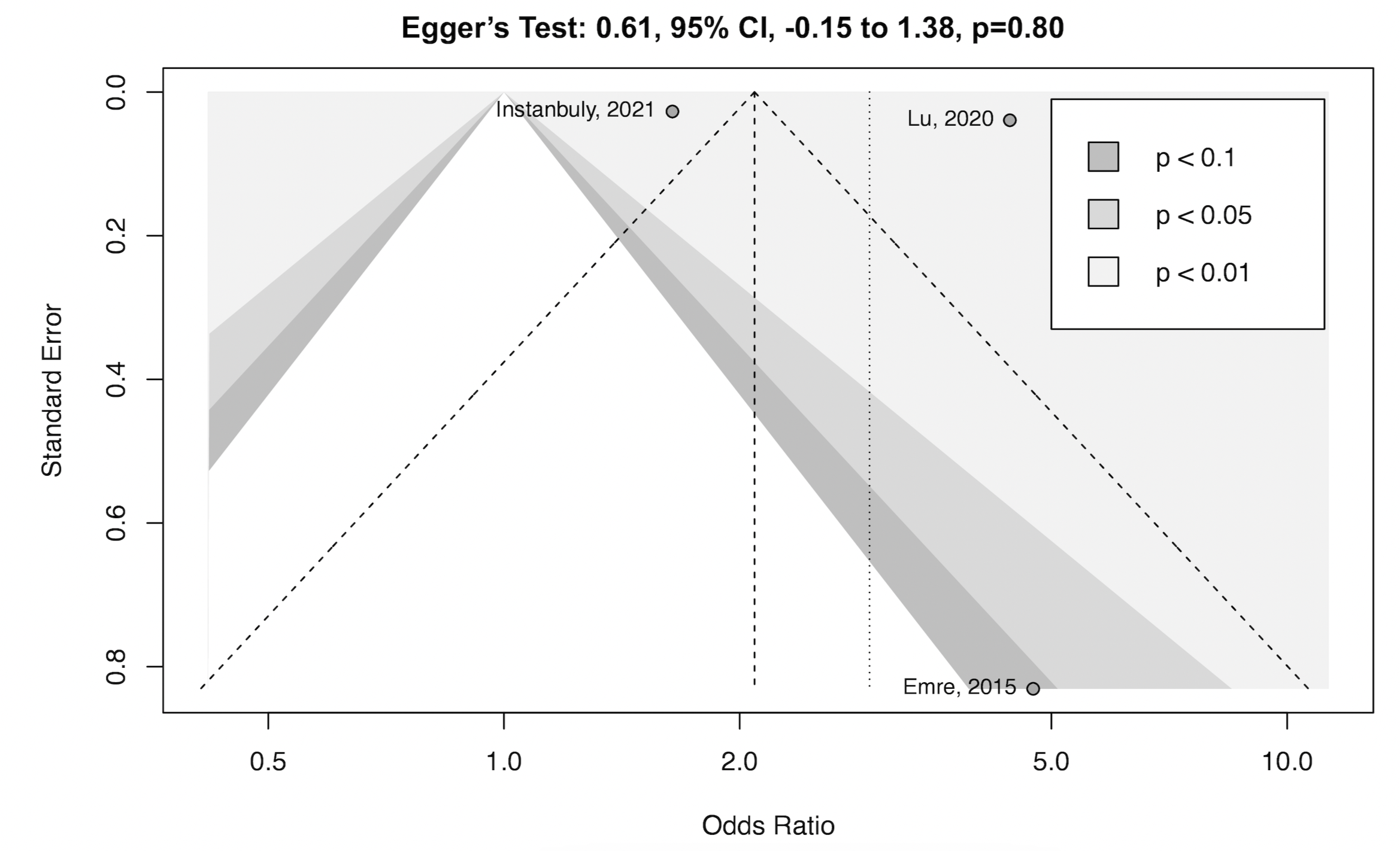
**
